# Natriuretic Hormone: The Ultimate Determinant of the Preservation of External Sodium Balance

**DOI:** 10.3389/fendo.2014.00212

**Published:** 2014-12-11

**Authors:** Neal S. Bricker, Christopher D. Cain, Stewart Shankel

**Affiliations:** ^1^School of Medicine, University of California at Los Angeles, Los Angeles, CA, USA; ^2^Phytoanalytics, Grand Terrace, CA, USA; ^3^Department of Medicine, School of Medicine, University of California at Riverside, Riverside, CA, USA

**Keywords:** natriuretic hormone, sodium transport, ENaC, xanthurenic acid derivatives, synthesized NH

## Abstract

The present manuscript focuses on a putative natriuretic hormone. It includes the history of a long-term search for the pure molecule, ranging from partial purification to synthesis. It includes a description of seven different bioassay systems used, a resume of the sequential steps in purification, and a summary of a series of experimental protocols employed in the effort to define the biologic properties of the inhibitor of sodium (Na) transport. Two closely related molecules were purified and synthesized. Both are xanthurenic acid derivatives (xanthurenic acid 8-O-β-D-glucoside and xanthurenic acid 8-O-sulfate). It is concluded that one or both of these two low molecular weight compounds (MW: 368 and 284) meet many of the criteria for the final modulator of Na excretion.

## Introduction

It is our assumption that there is a single natriuretic hormone that serves to modulate the renal excretion of sodium (Na) so as to preserve an ongoing equality between consecutive 24 h Na excretion by the kidneys and the contemporaneous intake of Na. The focus on what we presume to be a hormone was initiated from the classic experiments of DeWardener et al. (1–3). The present symposium provides a contemporary review of the state-of-the-art of natriuretic hormone research by key investigators who have pursued the identity and biologic properties of several different putative natriuretic hormones.

Because of the design of DeWardener’s protocol, it appeared likely that all of the factors then known to affect the renal excretion of Na could be excluded as the definitive control element. These included changes in glomerular filtration rate (GFR) (so-called “first factor”) and changes in mineralocorticoid hormone activity (“second factor”). We coined the term “third factor” (4), which subsequently was replaced by “natriuretic factor.”

Throughout a long period of time, a multinational group of investigators has sought to isolate and characterize the natriuretic hormone. To this point in time, despite considerable progress, the ultimate goal remains elusive. Hence, the present symposium.

Our interest in an endogenous natriuretic hormone arose out of long-term studies on the pathologic physiology of chronic progressive renal disease (CRD) (5). It was the pattern of Na excretion that evolved as nephron destruction proceeded.

What is a remarkable change in the estimated Na excretion rate per nephron from the beginning to the end of CRD is presented in Table 1. In constructing this table, the intake of Na per 24 h was maintained constant (e.g., at 120 mEq/day) and at all levels of GFR, external Na balance was preserved (the latter is not unusual down to a GFR as low as 10% of normal.).

**Table 1:** **Adaptation in Na excretion per nephron in advancing CRD on a constant salt intake**.

GFR ml/mn	Na in mEq/day	Na out mEq/day	μl SNGFR % of normal	Na out per nephron nEq	Magnification per nephron
120	120	120	100	700	1
60	120	120		1,400	2
30	120	120		2,800	4
15	120	120	200	5,600	8
7 1/2	120	120		11,212	16

*See text in reference to the increase in SNGFR. Column 6 depicts the exponential increase in sodium excretion per nephron per unit of sodium intake*.

At a normal GFR of 120 ml/min in an 80 kg person, Na balance is achieved by the excretion of 1/2 of 1% of the filtered load of Na. A fall in GFR to 60 ml/min mandates the excretion of 1% of the filtered Na. A further reduction of GFR (to 30 ml/min) due to the progression of the underlying renal disease requires the excretion of 2% of the filtered Na. And at a GFR of 15 ml/min, external Na balance is achieved by the excretion of 4% of the filtered Na. Thus, per unit of Na entering the extracellular fluid on a constant Na intake, the excretion rate per milliliter of residual GFR with no time delay increases by eightfold. Finally, in many forms of CRD, single nephron GFR (SNGFR) doubles as nephron loss advances. Na excretion rate per residual nephron per milliequivalents of Na intake, approaches 16 times the value in the normal subject.

## Bioassay Systems

Seven different bioassay systems were used in both the sequential steps in the isolation, purification, and synthesis of NH and the studies of the biologic effects of the test materials. Each assay system allowed for a quantitative measure of the inhibitory effects of a test sample on Na transport either *in vitro* or *in vivo*.

The primary *in vitro* systems (frog skin and toad bladder) (6, 7) involve polar epithelial cells that transport Na from the serosal surface of the membrane across the mucosal surface. Na transport is quantified by the short-circuit current across the isolated membrane (in an Ussing chamber). Activity of partially purified to pure NH was detectable only when the inhibitor was added to the serosal surface of the membrane.

In micropuncture studies (8), isolated cortical collecting tubules, dissected from normal rabbits were perfused with partially purified test material from the urine of uremic patients. The perfusate was delivered into the lumen of each nephron segment and the peritubular surface was bathed in a solution of known composition (see Experimental Data).

The *in vivo* assays were performed in rats (9). The test materials were delivered intravenously, intraarterially, or via a gastric tube.

In the rat assay, the rats employed had: (1) two normal kidneys; (2) one remnant kidney (75% reduction of renal mass) and a contralateral normal kidney; or (3) a solitary remnant kidney following removal of the normal kidney.

## Purification of NH

Twenty-four hour collections of urine were reduced from their original volume to approximately 20 ml of sludge by lyophilization. The sludge was then redissolved in isotonic saline to 25 ml components each equal to 6 h of original urine. Each sample then was chromatographed through Sephadex G-25 with online monitoring of UV absorption at 290 nm and electrical conductivity (10).

Biologic activity was limited to the “post-salt” peaks using the frog skin assay (11). Short-circuit current decreased from 40–50 to 20–30 μAmp/cm^2^. These samples were purified using high performance liquid chromatography (HPLC) runs. The active fractions were purified further by two consecutive HPLC runs. The eluate was monitored by fluorescence and UV absorbance.

After further concentration, a bioassay was performed by infusion of the test material into the uremic rat (11). A strong and sustained natriuresis was recorded.

The natriuretic fractions, consistently showed two peaks with strong fluorescence (excitation 332 nm; emission 430 nm) and characteristic UV absorption (UV max at 338 nm). These spectroscopic signatures were used as the main pooling criteria for further HPLC-based purification of NH (11). Additional steps in isolation, purification, and synthesis of the two molecules: xanthurenic acid 8-O-β-D-glucoside and xanthurenic acid 8-O-sulfate are described in a separate publication (11).

## Experimental Data

### Uremic vs. control subjects

Based on the adaptation in Na excretion in advancing CRD (see Table 1), bioassays were performed on normal rats using partially purified urine samples from 17 patients with advanced CRD (mean GFR 8.7 ml/min) and 14 normal control subjects. The assays from the normal subjects were negative [i.e., no significant increase in either absolute Na excretion (U_Na_V), or the fraction of filtered Na excreted (FE_Na_) (12)]. The uremic fractions produced a highly significant increase from baseline levels in both parameters of Na excretion. With more concentrated samples of the uremic urine fractions, values for ΔFE_Na_ rose to levels as high as 12%.

### Advanced CRD with a superimposed edema-forming state: (The nephrotic syndrome)

Eight patients with advanced CRD and the nephrotic syndrome were studied (12). Assays were performed on normal rats using partially purified fractions of serum in all eight studies. Fractions of urine were also used in three studies. Values for U_Na_V decreased from control levels (by an average of 0.97 μEq/min). The mean value for FE_Na_ also decreased (1.35%).

Danovitch et al. (13) studied a group of five patients with far advanced CRD (GFR 5.2–16.0 ml/min) who initially were in Na balance on controlled metabolic diets containing from 58 to 342 mEq of Na per day. In each patient, while under close observation (clinical and laboratory), the Na content of the diet was reduced at intervals of 1 week or longer over 4–14 weeks. Four of the patients exhibited a salt-losing state, wherein Na excretion exceeded Na intake. Two of these patients required intravenous salt replacement. At the completion of the studies, all patients maintained external Na balance while ingesting a mean of 5.0 ± 2.9 mEq of Na/day. Thus, in contrast to patients with advancing CRD who maintain Na balance on a constant salt intake and a progressively decreasing nephron population, these patients were subjected to a progressive reduction of Na intake with an unchanging nephron population. (GFR remained constant throughout the studies.) The adaptive increase in Na excretion in advancing CRD (Table 1), thus was reversed with slow (and cautious) serial reductions in salt intake. The salt-losing tendency of CRD was reversed.

### Micropuncture studies

As described under Bioassay Systems, partially purified natriuretic factor was infused into the lumen of isolated cortical collecting tubules of normal rabbits. No effect was observed with intraluminal infusions. However, when the natriuretic material was added to the solution bathing the peritubular surface, the effects were rapid in onset and highly significant. Net Na flux (measured isotopically) from luminar to peritubular surface of the nephrons, decreased from 6.29 to 3.20 pmol/s (*p* < 0.001) with no change in Na flux in the opposite direction. Potential difference rose rapidly from −22.5 to −12 mV. Control studies, using the same fraction from normal subjects, had no effect (8).

### Studies on normal human beings exposed to water immersion

Epstein et al. (14) have established water immersion to the neck as a reliable and reproducible stimulus evoking natriuresis in normal subjects. The experiments were performed on 12 normal adults (15). Each was studied twice – once under control conditions sitting in a chair and again during water immersion. The paired studies were performed at the same time of day. Partially purified urine samples were assayed for natriuretic activity in normal rats.

U_Na_V and FE_Na_ did not change from the baseline values in the control studies. During water immersion U_Na_V rose (1.27 ± 0.28 μEq/min) and FE_Na_ increased from pre-immersion baseline values by 1.29 ± 0.21%. Both changes were highly significant (*p* < 0.001) (15).

### Studies in Dogs

Normal dogs were maintained on a Na intake varying from 3 to 258 mEq/day, with and without fludrocortisone. Urine was partially purified and assayed for natriuretic effect in normal rats and for inhibition of Na transport in isolated toad bladders. In dogs on the 258 mEq Na diet and 0.2 mg fludrocortisone/day, both a statistically significant natriuretic response and inhibition of short-circuit current (*p* < 0.001) were observed. In dogs fed 3 mEq of Na per day with fludrocortisone, no significant effect was found in either assay system (16).

### End-organ responsiveness

The effects of nephron loss on the natriuretic response (9) to partially purified natriuretic factor were studied in three groups of rats, each on a normal salt diet.

1. Group 1: normal rats: intraarterial infusion into one renal artery produced a unilateral natriuresis.
2. Group 2: intraarterial infusion into the renal artery of a unilateral remnant kidney in rats with a contralateral normal kidney produced an increase in FE_Na_, which was equal bilaterally. Intravenous infusion of the natriuretic fraction also produced comparable increments of FE_Na_ in the remnant and the normal kidneys.
3. Group 3: in uremic rats with a solitary remnant kidney (no contralateral kidney), the intraarterial infusion of natriuretic factor produced an increase in FE_Na_ that was significantly greater than in remnant kidneys of group 2 rats or normal kidneys of group 1.

### Natriuretic response to synthesized (pure) NH

Synthesized preparations of xanthurenic acid 8-O-β-D-glucoside (NH) and xanthurenic acid 8-O-sulfate (NH-1) were bioassayed in normal rats (11).

The intravenous infusion of NH (range 0.14–16.4 nmol) and NH-1 (0.7–4.21 nmol) was studied in eight and five normal rats, respectively. In the NH group, ΔU_Na_V averaged 3.68 ± 0.55 μEq/min; in the five experiments in which NH-1 was the test substance, ΔU_Na_V averaged 4.33 ± 0.71 μEq/min (1).

In five additional studies, a combination of NH (1.4–3.41 nmol) and NH-1 (1.75–5.44 nmol) was assayed. ΔU_Na_V averaged 5.0 ± 0.89 μEq/min.

### Studies by Hoffman and associates

In a recent publication, Hoffman et al. (17) studied the effects of xanthurenic acid 8-O-β-D-glucoside on Na excretion in adult male Sprague-Dawley rats. Each rat was given two consecutive incremental doses (6.3 + 31.5 nmol) of NH (designated as XAG). Values for ΔU_Na_V were 3.21 ± 1.12 and 3.99 ± 0.95 at the two dosages, respectively. Values for ΔFE_Na_ increased significantly (1.63 ± 0.46) during the second dose of XAG.

In these studies, GFR (inulin clearance) remained unchanged. Mean arterial pressure (MAP) and total renal blood flow were recorded electronically every 5 min for 60 s during the control periods and for 30–40 min periods during the XAG infusions. All hemodynamic values remained stable (17).

Two important new observations were made by Hoffman and coworkers:

1. In rats pretreated with amiloride, an inhibitor of E_Na_C, the epithelial Na channel in the distal tubule, the natriuretic effects of XAG were completely abolished (17).
2. In rats subjected to chronic blockade of the NO system, the natriuretic response to XAG was diminished suggesting to these investigators that the renal effects of XAG could be mediated in part by activation of the renal NO system (17).

## Discussion

A large number of factors, both humoral and physical are known to influence the renal tubular transport of Na. But none of these, including changes in GFR or mineralocorticoid hormone activity (see Introduction) is believed to be the final modulator of net Na transport and thus of Na excretion. We believe that natriuretic hormone fulfills this role. It thus would serve as the definitive element of a sophisticated biologic control system that is charged with the preservation of Na balance and with the constancy of the extracellular fluid volume. But, while there may be virtual unanimity of opinion about the existence of natriuretic hormone, there is no such unanimity about the nature of this hormone.

In this manuscript, we have reviewed the properties of a natriuretic factor, which we have pursued for a long period of time. The activity was obtained from both serum (or plasma) and urine using material that ranged from partially purified to pure, chemically synthesized molecules (11). We believe that the experimental data reviewed in the manuscript meet at least some of the criteria for natriuretic hormone. These include:

1. The rapid and reversible inhibition of net Na transport across polar epithelial cell systems, including the distal portion of the nephron.
2. The foregoing biologic activity is present only when the inhibitor is added to the peritubular surface of the nephron or the serosal surface of equivalent *in vitro* models.
3. The natriuretic effect of the purified and synthesized material is completely blocked by prior administration of amiloride, an inhibitor of E_Na_C activity in the distal portion of the nephron.
  The mechanism of this action could relate either to a direct effect on the number and/or the activity of E_Na_C channels, or to a change in the relation between open vs. closed E_Na_C channels.
4. Inhibition of Na transport in the distal nephron associated with a rapid shift in transepithelial electric potential difference (i.e., a less negative intraluminal potential).
  This change would favor the excretion of Cl with Na as opposed to increased secretion of K.
5. No evidence for a fixed coupling ratio between the inhibition of Na transport and K secretion.
6. Increase in natriuretic activity in advancing CRD in purified serum or urine samples.
7. An increase in end-organ responsivity to the inhibitor associated with nephron loss.
8. Lack of natriuretic effect of the inhibitor in patients with advanced CRD and a superimposed edema-forming state (the nephrotic syndrome).
9. Reversal of salt-losing state in advanced CRD by progressive slow reduction of salt intake to very low levels.
10. Presence of natriuretic activity in urine of normal subjects during water immersion – an experience known to produce central hypervolemia.
11. Increased natriuretic activity in normal dogs on a high salt diet and superimposed mineralocorticoid hormone.
12. No natriuretic activity in normal dogs on a low salt diet and superimposed mineralocorticoid hormone.

## Other “Natriuretic” Factors

Four other categories of putative natriuretic hormones are considered in separate papers in this symposium. These compounds include: (1) ouabain [or ouabain-like substances (OLS)]; (2) marinobufogenin (MFG); (3) vanadium diascorbate (VD); and atrial natriuretic peptides (ANP).

A summary of key properties of each is shown in Table 2 and a brief description of some relevant characteristics follows.

**Table 2:** **A comparison of several natriuretic substances**.

	Ouabain (OLS)	Marinobufogenin (MFG)	Vanadium diascorbate (VD)	Atrial natriuretic peptides			Xanthurenic acid 8-O-β-D-glucoside (XAG)
				ANP	BNP	CNP	
Isolation	Plasma and adrenal cortex and hypothalamus (18–23)	Plasma, urine, and adrenal cortex (24, 25)	Urine and plasma (26)	Atria, heart, and kidney (27)	Brain, heart, and kidney (27)	Brain, heart, and vasculature (27, 28)	Plasma and urine from uremic patients (6, 8, 10, 29)
Stimulus	AII, ACTH, ↑BP positive sodium balance or intake; ↑serum K+ up to 5 mEq/l	Same as OLS	Salt loading, aldosteronism, and volume expansion (26)	Stretch of cardiac wall – especially the atria, ET adrenergic stimuli (30)	Same as ANP (30)	Same? as ANP (30)	Normal and uremic patients and animals (10, 15)
M.W.	584.6 (31)	387? 600? (31)	403	2,000–3,000±(32)	2,000–3,000±(32)	2,000–3,000±(32)	368 (glucoside) 284 (sulfate) (11)
Structure	Steroid (31)	Steroid (31)	Vanadate diascorbate from ascorbic acid (26)	28 AA (32)	32 AA (32)	22 AA (32)	8-O-(-D-glucoside 8-O-sulfate of xanthurenic acid from tryptophan (11)
Site of Action	α2α3 NaK ATPase primarily (18–23, 25, 26, 33–43) acts on basolateral membrane of PCT and NHE3 in PCT (36, 37)	α1 NaK ATPase in PCT (24, 44)	NaK ATPase in PCT acts on basolateral membrane(26)	Blocks ENaC, ↑GFR, blocks NaK ATPase AII in PCT (30, 45, 46) ↓H_2_O absorption in CT, ↓urine concentration (47, 48)	Similar to ANP	Direct vasodilator and simulator to ANP (47)	Blocks ENaC acts from basolateral surface (17)
Natriuresis	Variable from no natriuresis to mild natriuresis (21, 25, 33, 36, 38, 39)No effect on α1-NaK ATPase	Variable natriuresis (24, 44)	Moderate natriuresis (26)	Moderate through CGMP (28, 30, 32, 46)	Same as ANP(28, 30, 32, 46, 49)	None (28, 47)	Eliminated by blocking ENaC (17)
RBF	↓(40)	?	?	↑RBF → ↑GFR (30, 45, 50)	Variable (50)	No	No effect (17)
GFR	↓Or no change (40)	?	?	↑GFR dilates afferent arteriole and constricts efferent (49)	Same as ANP (49)	No	No effect (17)
K excretion	↓	↓	↓	↑	↑	↑	Minimal (11)
Vasoactivity	↑BP, vasoconstriction (21, 22, 33, 34, 36, 40–43) ↑Ca influx and Na influx into vessel wall (22, 34, 42–44)	↑BP, vasoconstriction (24, 44)	↑BP (27) ↑Ca and Na influx into vessel wall (25, 27)	↓BP (32, 47)	↓BP (32, 47)	?	None (17)

### Ouabain (OLS)

A small molecule (MW 584.6) isolated from plasma (18), urine, adrenal cortex, and hypothalamus (19–23). The primary and relevant effects are the inhibition of NaK ATPase and the cross reaction with ouabain antibodies (21).

But they are inconsistently natriuretic (21, 22, 33, 34), presumably because ouabain has little effect on the α1 subunit of NaK ATPase (22, 35). Indeed, there also recent evidence that OLS may actually cause Na retention (26).

### Marinobufogenin

Also a small molecule (MW between 387 and 600) (31), which is isolated from plasma, urine, and the adrenal cortex (24, 44). No studies have shown that administration of MFG causes natriuresis in assay animals. However, administration of anti-MBG antibodies reduces Na excretion (24, 44). Exogenous administration of bufalin, a very closely related compound, injected into the renal artery of sheep has been shown to produce natriuresis. No similar studies have as yet been published using MFG (51).

### Vanadium diascorbate

A small molecule (MW 403) isolated from plasma and urine (26). Inhibits NaK ATPase in the proximal convoluted tubule. Produces moderate natriuresis, may produce influx of Ca^++^ into vessel walls and thereby increase BP.

### Atrial natriuretic peptides

Molecular weight 2,000–3,000± (32). The natriuretic peptides, while producing natriuresis, also increase GFR and increase glomerular pressure by dilating the afferent arteriole, and constricting the efferent arteriole (30, 45, 50). It may cause progressive renal failure in animal models.

If ANP is the final modulator of Na balance, there is a paradox. In advanced cirrhosis, there is an increase in preload and a decrease in central volume and EAV. ANP levels are elevated (50). Likewise in CHF there is a decrease in EAV and an increase in preload and again ANP levels are elevated. Physiologically both situations should invoke Na retention due to the decrease in the EAV, which in turn should shut off natriuresis, but in both of these situations ANP is elevated.

The arterial natriuretic peptides are primarily vasoactive and act on multiple sites of the nephron including the proximal tubule.

## Conclusion

The cumulative data presented in this paper lend support to the view that xanthurenic acid 8-O-β-D-glucoside and xanthurenic acid 8-O-sulfate could be the long sought after and elusive natriuretic hormone. But to validate, or refute this thesis, additional experimental evidence is required. A partial list of key areas of future study includes:

1. A sensitive assay system for quantifying the levels of the putative hormone in body fluids.
2. Establishing the site of production.
3. The character of the signaling process (including the nature of the receptors), involved in initiating and controlling the induced natriuresis.
4. The enzymes involved in the synthesis.

## Conflict of Interest Statement

During the long period of study covered by this review, major support was provided by Program Project Grants from the NIH and from Naturon Pharmaceutical Company. The authors do not have any conflict of interest in relation to this review.

## References

[1] de Wardener HE Mills IH Clapham WF Hayter CJ Studies on the efferent mechanism of the sodium diuresis which follows the administration of intravenous saline in the dog. Clin Sci (1961) 21:249–58. 13884596

[2] Mills IH de Wardener HE Hayter CJ Clapham WF Studies on the afferent mechanisms of the sodium diuresis which follows the administration of saline in the dog. Clin Sci (1961) 21:259–64. 14474186

[3] Nutbourne DM de Wardener HE The effect of a water diuresis on the urinary excretion hydrogen ions in man. Clin Sci (1961) 20:63–73. 13729936

[4] Bricker NS The control of sodium excretion with normal and reduced nephron populations. The pre-eminence of third factor. Am J Med (1967) 43:313–21 10.1016/0002-9343(67)90188-X 5341544

[5] Bricker NS Zea L Shapiro M Sanclemente E Shankel S. Biologic and physical characteristics of the non-peptidic, non-digitalis-like natriuretic hormone. Kidney Int (1993) 44:937–47. 10.1038/ki.1993.335 8264153

[6] Bourgoignie J Klahr S Bricker NS. Inhibition of transepithelial sodium transport in the frog skin by a low molecular weight fraction of uremic serum. J Clin Invest (1971) 50:303–11. 10.1172/JCI106495 5540168 PMC291924

[7] Buckalew VM Martinez FJ Green WE. The effect of dialysates and ultrafiltrates of plasma of saline-loaded dogs on toad bladder sodium transport. J Clin Invest (1970) 49:926–35. 10.1172/JCI106312 5441546 PMC535765

[8] Fine LG Bourgoignie JJ Hwang KH Bricker NS. On the influence of the natriuretic factor from patients with chronic uremia on the bioelectric properties and sodium transport of the isolated mammalian collecting tubule. J Clin Invest (1976) 58:590–7. 10.1172/JCI108505 956387 PMC333217

[9] Fine LG Bourgoignie JJ Weber H Bricker NS. Enhanced end-organ responsiveness of the uremickidney to the natriuretic factor. Kidney Int (1976) 10:364–72. 10.1038/ki.1976.122 1003727

[10] Bricker NS Klahr S Purkerson M Schultze RG Avioli LV Birge SJ *In vitro* assay for a humoral substance present during volume expansion and uraemia. Nature (1968) 219:1059–1059 10.1038/2191058a0 5673365

[11] Cain CD Schroeder FC Shankel SW Mitchnick M Schmertzler M Bricker NS. Identification of xanthurenic acid 8-O-β-D-glucoside and xanthurenic acid 8-O-sulfate as human natriuretic hormones. Proc Natl Acad Sci U S A (2007) 104:17873–8. 10.1073/pnas.0705553104 17978185 PMC2077034

[12] Bourgoignie JJ Hwang KH Ipakchi E Bricker NS. The presence of a natriuretic factor in urine of patients with chronic uremia. The absence of the factor in nephroticuremic patients. J Clin Invest (1974) 53:1559–67. 10.1172/JCI107706 4830222 PMC302651

[13] Danovitch GM Bourgoignie JJ Bricker NS. Reversibility of the “salt-losing” tendency of chronic renal failure. N Engl J Med (1977) 296:14–9. 10.1056/NEJM197701062960104 618364

[14] Epstein M Renal effects of head-out water immersion in man: implications for an understanding of volume homeostasis. Physiol Rev (1978) 58:529–81. 356067 10.1152/physrev.1978.58.3.529

[15] Epstein M Bricker NS Bourgoignie JJ Presence of a natriuretic factor in urine of normal men undergoing water immersion. Kidney Int (1978) 13:152–8 10.1038/ki.1978.22 713275

[16] Favre H Hwang KH Schmidt RW Bricker NS Bourgoignie JJ. An inhibitor of sodium transport in the urine of dogs with normal renal function. J Clin Invest (1975) 56:1302–11. 10.1172/JCI108206 1184751 PMC301993

[17] Hoffman A Ovcharenko E Karram T Okun-Gurevich M Goltzman I Cain C Renal effects of a novel endogenous natriuretic agent xanthurenic acid 8-O-β-D-glucoside in rats. Physiol Rep (2013) 1:e00155. 10.1002/phy2.155 24400157 PMC3871470

[18] Hamlyn JM Blaustein MP Bova S DuCharme DW Harris DW Mandel F Identification and characterization of a ouabain-like compound from human plasma. Proc Natl Acad Sci U S A (1991) 88:6259–62. 10.1073/pnas.88.21.9907-d 1648735 PMC52062

[19] Dvela M Rosen H Ben-Ami HC Lichtstein D. Endogenous ouabain regulates cell viability. Am J Physiol Cell Physiol (2012) 302:C442–52. 10.1152/ajpcell.00336.2011 22031604

[20] Tamura M Lam TT Inagami T. Specific endogenous Na^+^, K^+^-ATPase inhibitor purified from bovine adrenal. Biochem Biophys Res Commun (1987) 149:468–74. 10.1016/0006-291X(87)90391-3 2827643

[21] Nesher M Dvela M Igbokwe U Rosen H Lichtstein D. Physiological roles of endogenous ouabain in normal rats. Am J Physiol (2009) 297:H2026–34. 10.1152/ajpheart.00734.2009 19837951

[22] Blaustein MP Leenen FHH Chen L Golovina VA Hamlyn JM Pallone TL How NaCl raises blood pressure: a new paradigm for the pathogenesis of salt-dependent hypertension. Am J Physiol (2011) 302:H1031–49. 10.1152/ajpheart.00899.2011 22058154 PMC3311458

[23] Tamura M Konishi F Sakakibara M Inagami T Large scale purification of an endogenous Na^+^/K^+^ -pump inhibitor from bovine adrenal glands. In: Bamberg E Schoner W, editors. Sodium Pump: Structure, Mechanism, Hormonal Control and its Role in Disease. New York, NY: Springer (1994). p. 763–6.

[24] Haller ST Kennedy DJ Shidyak A Budny GV Malhotra D Fedorova OV Monoclonal antibody against marinobufagenin reverses cardiac fibrosis in rats with chronic renal failure. Am J Hypertens (2012) 25:690–6. 10.1038/ajh.2012.17 22378033 PMC3355226

[25] Nowicki S Enero MA de Lores Arnaiz G. Diuretic and natriuretic effect of a brain soluble fraction that inhibits neuronal Na^+^, K^+^-ATPase. Life Sci (1990) 47:1091–8. 10.1016/0024-3205(90)90167-P 2172678

[26] Kramer HJ Krampitz A Bäcker A Meyer-Lehnert H. Ouabain-like factors in human urine: identification of a Na-K-ATPase inhibitor as vanadium-diascorbate adduct. Clin Exp Hypertens (1998) 20:557–71. 10.3109/10641969809053234 9682912

[27] Beltowski J Wójcicka G. Regulation of renal tubular sodium transport by cardiac natriuretic peptides: two decades of research. Med Sci Monit (2002) 8:RA39–52. 11859295

[28] Stingo AJ Clavell AL Aarhus LL Burnett JC Jr. Cardiovascular and renal actions of C-type natriuretic peptide. Am J Physiol (1992) 262:H308–12. 1310235 10.1152/ajpheart.1992.262.1.H308

[29] Bourgoignie JJ Hwang KH Espinel C Klahr S Bricker NS. A natriuretic factor in the serum of patients with chronic uremia. J Clin Invest (1972) 51:1514–27. 10.1172/JCI106948 5024044 PMC292289

[30] Brenner BM Ballerman BJ Gunning ME Zeidel ML Diverse biological actions of atrial natriuretic peptide. Physiol Rev (1990) 70:665–99. 2141944 10.1152/physrev.1990.70.3.665

[31] Puschett JB Agunanne E Uddin MH. Emerging role of bufadienolides in cardiovascular and kidney diseases. Am J Kidney Dis (2010) 56:359–70. 10.1053/j.ajkd.2010.01.023 20417001

[32] Lee CYW Burnett JC Natriuretic peptides and therapeutic applications. Heart Fail Rev (2007) 12:131–42 10.1007/s10741-007-9016-3 17440808

[33] Manunta P Messaggio E Ballabeni C Sciarrone MT Lanzani C Ferrandi M Plasma ouabain-like factor during acute and chronic changes in sodium balance in essential hypertension. Hypertension (2001) 38:198–203. 10.1161/01.HYP.38.2.198 11509476

[34] Anderson DE Fedorova OV Morrell CH Longo DL Kashkin VA Metzier JD Endogenous sodium pump inhibitors and age-associated increases in salt sensitivity of blood pressure in normotensives. Am J Physiol (2008) 294:R1248–54. 10.1152/ajpregu.00782.2007 18287222 PMC2430746

[35] Katz A Lifshitz Y Bab-Dinitz E Kapri-Pardes E Goldschleger R Tal DM Sensitivity of digitalis glycosides for isoforms of human Na,K-ATPase. J Biol Chem (2010) 285:19582–92. 10.1074/jbc.M110.119248 20388710 PMC2885237

[36] Liu J Xie Z-J. The sodium pump and cardiotonic steroids-induced signal transduction protein kinases and calcium-signaling microdomain in regulation of transporter trafficking. Biochim Biophys Acta (2010) 1802:1237–45. 10.1016/j.bbadis.2010.01.013 20144708 PMC5375027

[37] Khundmiri SJ Advances in understanding the role of cardiac glycosides in control of sodium transport in renal tubules. J Endocrinol (2014) 222(1):R11–24 10.1530/JOE-13-0613 24781255

[38] Gupta S Yan Y Malhotra D Liu J Xie Z Najjar SM Ouabain and insulin induce sodium pump endocytosis in renal epithelium. Hypertension (2012) 59:665–72. 10.1161/HYPERTENSIONAHA.111.176727 22311908 PMC3336087

[39] Periyasamy SM Liu J Tanta F Kabak B Wakefield B Malhotra D Salt loading induces redistribution of the plasmalemmal Na/K-ATPase in proximal tubule walls. Kidney Int (2005) 67:1868–77. 10.1111/j.1523-1755.2005.00285.x 15840034

[40] Nechay BR Chinoy DA Effect of ouabain on renal transport of P-aminohippuric acid (PAH) and blood flow in the dog. Eur J Pharmacol (1968) 3:322–9 10.1016/0014-2999(68)90115-5 5674650

[41] Liu J Yan Y Liu L Xie Z Malhotra D Joe B Impairment of Na/K-ATPase signaling in renal proximal tubule contributes to Dahl salt-sensitive hypertension. J Biol Chem (2011) 286:22806–13. 10.1074/jbc.M111.246249 21555512 PMC3123048

[42] Zhang J Lee MY Cavalli M Chen L Berra-Romani R Balke CW Sodium pump α2 subunits control myogenic tone and blood pressure in mice. J Physiol (2005) 569:243–56. 10.1113/jphysiol.2005.091801 16166162 PMC1464198

[43] Blaustein MP Zhang J Chen L Song H Raina H Kinsey SP The pump, the exchanger, and endogenous ouabain. Signaling mechanisms that link salt retention to hypertension. Hypertension (2008) 53:291–8 10.1161/HYPERTENSIONAHA.108.119974 19104005 PMC2727927

[44] Fedorova OV Talan MI Agalakova NI Lakatta EG Bagrov AY. Endogenous ligand of α_1_ sodium pump, marinobufagenin, is a novel mediator of sodium chloride-dependent hypertension. Circulation (2002) 105:1122–7. 10.1161/hc0902.104710 11877366

[45] Light DB Corbin JD Stanton BA. Dual ion channel regulation by cyclic GMP and cyclic-dependent protein kinase. Nature (1990) 344:336–9. 10.1038/344336a0 1690355

[46] Valdivieso A. The kidney in chronic liver disease: circulatory abnormalities, renal sodium handling and role of natriuretic peptides. Biol Res (1998) 31:291–304. 9830517

[47] Weidmann P Hasler L Gnadinger MP Lang RE Uehlinger DE Shaw S Blood levels and renal effects of atrial natriuretic peptide in normal man. J Clin Invest (1986) 77:734–42. 10.1172/JCI112368 2936762 PMC423457

[48] Pandey KN Biology of natriuretic peptides and their receptors. Peptides (2005) 26:901–32 10.1016/j.peptides.2005.03.055 15911062

[49] Eiskjaer H Pedersen EB. Dose-response study of atrial natriuretic peptide bolus injection in healthy man. Eur J Clin Invest (1993) 23:37–45. 10.1111/j.1365-2362.1993.tb00715.x 8383057

[50] Gunning ME Brenner BM. Natriuretic peptides and the kidney: current concepts. Kidney Int (1992) 42:S127–33. 1405363

[51] Yates NA McDougall IG. Effect of direct arterial infusion of bufalin and ouabain in conscious sheep. Br J Pharmacol (1993) 108:627–30. 10.1111/j.1476-5381.1993.tb12852.x 8385530 PMC1908056

